# Acquired Inhibitors: A Special Case of Bleeding in Older Adults

**DOI:** 10.1155/2012/308109

**Published:** 2012-11-29

**Authors:** Richard G. Stefanacci

**Affiliations:** Department of Health Policy & Public Health, University of the Sciences in Philadelphia, 600 South 43rd Street, Philadelphia, PA 19104, USA

## Abstract

This literature review is intended to familiarize physicians and healthcare providers of older adults with the potential causes of acute bleeding in older adults and to review diagnostic approaches that can produce prompt identification of acute bleeding and facilitate timely treatment. Adverse events from anticoagulant treatment and nonsteroidal anti-inflammatory drug (NSAID) and aspirin use and abuse are among the most common causes of bleeding in older adults. Diagnoses infrequently considered—mild congenital hemophilia, acquired hemophilia, von Willebrand disease, and platelet dysfunction—can contribute to acute bleeding in older adults. The approach to management of bleeding varies. Management of acute bleeding in older adults can be challenging because these patients often have chronic comorbidity and have been prescribed long-term concomitant medications that can complicate diagnosis and treatment. Prompt recognition of acquired hemophilia, referral to an expert hematologist, and timely initiation of treatment could improve outcome in older patients who experience bleeding episodes resulting from this condition.

## 1. Introduction

The diagnosis of acute bleeding in older adults is challenging because the presence of chronic comorbidities and polypharmacy not only places these individuals at increased risk for bleeding, but also complicates the identification of underlying causes [1, 2]. While adverse events from anticoagulant treatment and nonsteroidal anti-inflammatory drug (NSAID) and aspirin use and abuse are among the most common causes of bleeding in older adults [3–9], other less frequent etiologies should not be overlooked [1, 2]. Diagnoses not often considered—such as previously undiagnosed mild congenital hemophilia, acquired hemophilia, von Willebrand disease, and platelet dysfunctions associated with uremia and liver cirrhosis—can all contribute to acute bleeding in older adults. 

Acquired factor VIII inhibitors (acquired hemophilia) are a rare but potentially life-threatening cause of acute bleeding in older adults [10–12], with fatal bleeding occurring in an estimated 21% of patients [12]. Because of its rarity, acquired hemophilia is often not considered in the differential diagnosis. This failure to recognize acquired hemophilia often results in delayed initiation of appropriate treatment, and misdiagnosis, with initiation of potentially harmful procedures and/or treatments, can negatively affect patient outcome [13–15]. Surgery in patients with acquired hemophilia can be particularly problematic [15], especially when performed without knowledge of the presence of acquired hemophilia and without appropriate preoperative inhibitor elimination therapy. 

Because older adults, especially those residing in nursing homes, are often in poorer health than their community-dwelling counterparts, with high rates of comorbidity and medication use [16], identifying the causes of acute bleeding in this population may be particularly complex. The objectives of this paper are to familiarize physicians and healthcare providers of older adult patients with the potential causes of acute bleeding in older adults and to review diagnostic approaches that can bring about the prompt identification of acute bleeding and, therefore, facilitate timely treatment. Because acquired hemophilia is rarely discussed and often overlooked as a potential cause of bleeding in older adults, the differential diagnosis and management of acquired hemophilia will be discussed in detail. 

## 2. Common Causes of Bleeding in Older Patients

Common causes of bleeding, especially in older adults, are seen because of complications of anticoagulant treatment [8, 17, 18]. The presence of comorbid conditions and polypharmacy may contribute to this increased risk, as may instability of therapeutic control caused by poor adherence to prescribed therapy. In a report reviewing patient data from January 2004 through December 2005, by the Centers for Disease Control and Prevention (CDC), one of the three drugs most commonly associated with emergency room/department visits was warfarin [19]. Other possible causes of bleeding in older patients include treatment with heparin, aspirin therapy, and NSAID use or abuse. Patients experiencing bleeding while receiving oral anticoagulant therapy may require subcutaneous or intravenous vitamin K administration. Fresh frozen plasma may also be administered when bleeding is severe. Patients experiencing bleeding while receiving heparin may require protamine sulfate administration [8, 18]. Platelet transfusions are sometimes used to restore platelet function [8]. Bleeding associated with aspirin use is often managed conservatively with local hemostatic procedures [18], and gastrointestinal bleeding associated with NSAIDs can usually be managed by cessation of the NSAID plus short-term administration of a proton pump inhibitor [20].

## 3. Overview of Acquired Hemophilia

### 3.1. Epidemiology

Acquired hemophilia is an autoimmune condition characterized by acute bleeding [21] that arises from the development of autoantibodies directed against clotting factors, most commonly factorVIII [21]. This disorder is rare: an estimated 1 to 4 persons per million are diagnosed with acquired factor VIII inhibitors each year [21]. The condition often goes unrecognized; as a result, the true incidence of acquired hemophilia is likely higher [22]. Older adults are particularly susceptible to acquired hemophilia, as is reflected in the higher reported incidence in this age group (14.7/million/year in the older adult population, 85 years of age or older) [10]. Underlying conditions such as autoimmune disorders, respiratory diseases, drug reactions, and malignancy are commonly present, identified in approximately half (or more) of cases [22].

### 3.2. Clinical Presentation

The clinical presentation of acquired hemophilia is different from that of congenital hemophilia. Most patients have no history of major bleeding and no family history of acquired hemophilia [14, 22]. A typical patient with acquired hemophilia is an older adult (median age, 77 years [13]) with recent-onset or acute bleeding. Younger individuals can also develop factor VIII inhibitors, particularly women during the postpartum period [10]. The majority of bleeding episodes in older adult patients are spontaneous or secondary to a trivial injury or minor invasive procedure, such as intramuscular injection or venous catheter insertion [13, 14, 23]. Only 25% of acute bleeding episodes are associated with a major inciting event such as surgery [14]. Bleeding is often severe, and 65.5% to 90% of affected patients present with a major bleeding event [10, 14]. 

Approximately 80% of patients with antifactor VIII inhibitors initially present with bleeds involving the skin, muscles, other soft tissues, mucosal surfaces of the nasal, gastrointestinal, or genitourinary tracts [13, 22]. Hemarthrosis, a hallmark of congenital factor VIII deficiency, is rare in acquired hemophilia [10, 24]. Retropharyngeal, retroperitoneal, and intracranial hemorrhages are also rare but serious consequences of acquired hemophilia and are associated with high rates of morbidity and mortality [24]. In a 2010 surveillance study conducted in the UK, gastrointestinal bleeding and bleeding in the lungs were the predominant causes of death within the first week after diagnosis, and retroperitoneal and intracranial hemorrhages and other soft tissue bleeds were the predominant causes of death beyond this period [24].

### 3.3. Diagnosis

Timely identification of acquired hemophilia may improve outcomes by facilitating prompt implementation of appropriate therapy. Other potential causes of bleeding must be ruled out through a review of the medical history and appropriate laboratory testing. This differential diagnosis should include mild to moderate hereditary hemophilia, lupus anticoagulant, bleeding complications of anticoagulant treatments, trauma, NSAID abuse, and other acquired bleeding disorders (such as acquired von Willebrand disease and acquired platelet dysfunction), in addition to acquired factor VIII deficiency [1]. 

**Figure 1:**
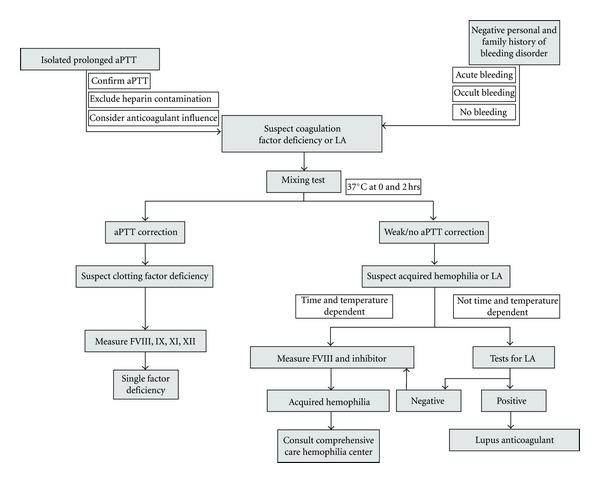
Algorithm to guide the management of patients with suspected acquired hemophilia. AH, acquired hemophilia; LA, lupus anticoagulant; F, coagulation factor; aPTT, activated partial thromboplastin time. Reprinted with permission from Collins et al. [13].

An isolated prolonged activated partial thromboplastin time (aPTT) should raise the suspicion of acquired hemophilia because the condition is characterized by prolonged aPTT in the absence of prothrombin time and platelet function abnormalities [11, 13]. Isolated aPTT prolongation, when present, should be further investigated using mixing tests, which can help determine whether the abnormality reflects a factor deficiency or the presence of an inhibitor (Figure 1) [11, 13]. If a correction of aPTT of less than 50% is observed after incubation with pooled normal plasma (1 : 1 ratio) from 1 to 2 hours, the presence of inhibitors should be suspected [13]. Further investigations should be performed to rule out heparin effect (history) and lupus anticoagulant (specific laboratory test) [11, 13]. 

### 3.4. Prognosis

The consequences of acquired hemophilia are potentially life threatening, and the reported overall mortality rate in a 2009 meta-analysis of data from 32 studies (377 patients) was 21% [12]. Mortality among older patients may be even higher (31% to 77%) [25, 26], possibly a reflection of overall poorer health and the presence of severe comorbid conditions [12, 25]. Delay in diagnosis and treatment also likely contribute to poor outcome in these patients [13, 14].

### 3.5. Management

Patients with acquired hemophilia will not respond to conventional treatment algorithms for bleeding, and referral to a hematologist is recommended to facilitate prompt initiation of appropriate treatment and to optimize long-term patient management [13]. Because of the acute needs of older adults with bleeding, referral to a hematologist is most likely to occur after an emergency room visit. The primary goal of acute therapy is the restoration of hemostasis, followed by the eradication of the factor VIII inhibitor [11, 13, 21].

#### 3.5.1. Restoring Hemostasis

Antihemorrhagic treatment may not be necessary in all cases (e.g., ecchymosis or subcutaneous hematoma) but should be initiated promptly in patients with active severe bleeding [11]. Historically, treatment selection was based predominantly on bleed severity and inhibitor titer [22, 27]. For inhibitor titers <5 Bethesda units (BUs)/mL in the absence of severe bleeding, high doses of factor VIII concentrate were recommended (to overwhelm the inhibitor) [22, 27]. Bypassing agents, which circumvent the role of the neutralized coagulation factor, were reserved for the treatment of patients with inhibitor titers ≥5 BU/mL or with severe bleeding. More recently, bypassing agents have been recommended as first-line therapy for all patients with acquired hemophilia who have any active bleeding [11, 13, 21]. 

Commercially available bypassing agents include recombinant activated factor VII (rFVIIa [NovoSeven RT]; Novo Nordisk A/S, Bagsværd, Denmark) [28] and plasma-derived activated prothrombin complex concentrate (pd-aPCC; factor eight inhibitor bypassing agent [FEIBA] NF; Baxter Healthcare Corporation, Westlake Village, CA) [29]. The hemostatic benefits of these agents have been documented in the published literature and are reviewed extensively elsewhere [11, 21]. The relative benefits and risks of these agents are unknown because no head-to-head clinical comparisons have been performed. The room temperature formulation of rFVIIa offers some practical advantages that may facilitate timely treatment, including room temperature stability prior to and for up to 3 hours after reconstitution, rapid dissolution, and small infusion volume [28]. Clinical trials in acquired hemophilia revealed that the incidence of thromboembolic events possibly or probably related to rFVIIa was 4%; however, the incidence in the elderly population has not been established [28]. High doses and use of antifibrinolytic agents within 12 hours of administration of pd-aPCC have been associated with an increased risk of thromboembolic events [29]. 

#### 3.5.2. Inhibitor Eradication

Because spontaneous remission is uncommon and unpredictable, all patients with acquired hemophilia should receive immunosuppressive therapy to eradicate factor VIII inhibitors and reduce the risk for future bleeding and associated morbidity and mortality [11, 13, 21]. Corticosteroids, either administered alone or in combination with cyclophosphamide, are recommended as first-line therapy and should be initiated at diagnosis and continued from 4 to 6 weeks [11, 13, 21]. Rituximab, an anti-CD20 monoclonal antibody, usually considered second-line therapy [11, 13, 21], may be used as first-line therapy when the potential side effects of immunosuppressive regimens (e.g., neutropenia) are of concern [11, 13, 21]. Immunosuppressive therapy-induced neutropenia may be particularly problematic in older adult patients, who commonly have comorbid medical conditions and may be at increased risk for infection. Rituximab is typically administered for up to 4 weeks [11, 21], with evidence of response usually present within 2 weeks [21]. Successful inhibitor eradication has also been reported with the use of other cytotoxic drugs (e.g., azathioprine, vincristine, mycophenolate, or cyclosporine) either alone or in combination with corticosteroids. These agents are typically reserved for patients who do not respond to first- or second-line therapy [11, 13, 21]. Immune tolerance induction is generally reserved for patients with life-threatening bleeding because experience with this approach in acquired hemophilia is limited [11].

## 4. Other Rare Causes of Bleeding in Older Patients

Mild to moderate hereditary hemophilia can contribute to bleeding in older patients and should not be disregarded as a potential cause of acute bleeding even without a diagnosis of congenital hemophilia because occasionally mild congenital hemophilia goes undiagnosed throughout adulthood [30]. In patients with mild-to-moderate congenital hemophilia and minor bleeding, the attainment of hemostasis is usually attempted using desmopressin or tranexamic acid [31]. Factor VIII concentrates are typically used if major bleeding is present. Bypassing agents should be used if factor VIII inhibitors are detected. 

Management of bleeding associated with other acquired bleeding disorders is directed at controlling acute bleeding and correction of the underlying abnormality. Management of acute bleeding in patients with acquired von Willebrand disease may include the administration of desmopressin, purified concentrates containing von Willebrand factor, and/or intravenous immunoglobulin [32]. Plasma exchange and extracorporeal immunoadsorption may be used to remove anti-von Willebrand factor antibodies. Treatment of underlying associated comorbidities (e.g., systemic lupus erythematosus, hypothyroidism, or malignancy) may facilitate permanent reversal of the bleeding disorder. Treatment of coagulopathy in patients with liver disease is aimed at replacement of deficient clotting factors and may include administration of fresh frozen plasma, cryoprecipitate, prothrombin complex concentrate, rFVIIa, or platelets [33]. Platelet transfusion is typically reserved for patients with active persistent bleeding and low platelet count (<50,000/mm^3^). Desmopressin may be administered to accelerate hemostasis in some patients and is sometimes used to control bleeding during invasive procedures [34]. Last, hemodialysis or peritoneal dialysis is typically used to manage platelet dysfunction in uremic patients [34]. Other potentially useful therapies include red blood cell transfusion and administration of erythropoietin, desmopressin, cryoprecipitate, estrogens, or recombinant erythropoietin [34, 35]. Specialist consultation and referral can ensure that patients with these conditions receive therapy appropriate for their specific acquired hemostatic abnormality.

Another relatively rare cause of bleeding in older adults that must be considered is the result of lupus anticoagulants. Lupus anticoagulants may underlie prolonged aPTT in some patients [11, 13] and may be associated with autoimmune diseases (including rheumatoid arthritis), infections, malignancy, or the use of certain medications (e.g., chlorpromazine, procainamide, quinidine, quinine, and antibiotics) [36, 37]. Bleeding associated with lupus anticoagulants is rare, as it is most commonly observed in pediatric patients [36]. Management typically targets any underlying disorder and correction of associated clinical abnormalities. Corticosteroids can be used to suppress these antibodies and restore the normal hemostatic process, if necessary.

## 5. Conclusions

The management of acute bleeding in older adults can be challenging because these patients often have chronic comorbid conditions and long-term concomitant medications that can complicate diagnosis and treatment. The differential diagnosis of older patients who experience a bleeding event should include etiologies such as acquired hemophilia, a rare and, therefore, often overlooked cause of bleeding in older adults. When the diagnosis of acquired hemophilia is missed, the outcome is often fatal. Prompt recognition of acquired hemophilia and timely initiation of treatment could improve outcome in older patients who experience bleeding episodes resulting from this condition.
